# Detection of Dengue Virus From *Aedes aegypti* (Diptera, Culicidae) in Field-Caught Samples From Makkah Al-Mokarramah, Kingdom of Saudi Arabia, Using RT-PCR

**DOI:** 10.3389/fpubh.2022.850851

**Published:** 2022-06-09

**Authors:** Elfadol Obeid Mohamed Ali, Ahmed Omer Babalghith, Adil Omer Saeed Bahathig, Ommer Mohamedelhassan Dafalla, Ibrahim Wasal Al-Maghamsi, Nasr Eldien Ali Gaafar Mustafa, Abdullah Ahmad Abdullah AL-Zahrani, Sameer Mohammed Yousef Al-Mahmoudi, Mohamed E. Abdel-Latif

**Affiliations:** ^1^Faculty of Public Health and Health Informatics, Umm Al-Qura University, Makkah, Saudi Arabia; ^2^Faculty of Medicine, Umm Al-Qura University, Makkah, Saudi Arabia; ^3^National Centre for Vectors Control Borne Diseases, Ministry of Health, Jazan, Saudi Arabia; ^4^Vectors Control Department, Ministry of Health, Makkah, Saudi Arabia; ^5^Department of Neonatology, Centenary Hospital for Women and Children, Canberra Hospital, Canberra, ACT, Australia; ^6^Department of Public Health, La Trobe University, Melbourne, VIC, Australia; ^7^Discipline of Neonatology, The Medical School, College of Health and Medicine, Australian National University, Acton, ACT, Australia

**Keywords:** larval ecology, Makkah, dengue fever, dengue virus, vectors, *Aedes*

## Abstract

Dengue fever (DF) is endemic to Makkah and Jeddah, the Kingdom of Saudi Arabia (KSA). However, until recently, the circulation of dengue virus (DENV) in *Aedes* mosquitoes in these areas was unknown. Serological surveillance of DENV in *Ae aegypti* is a powerful tool for early detection of dengue outbreaks and essential for developing effective control strategies. Therefore, this research aimed to examine a sample of adult *Ae aegypti* mosquitoes from Makkah, KSA, to detect DENV. In total, 1295 *Ae aegypti* mosquitoes were collected from the field from target areas of Makkah with a high incidence and prevalence of DF. The samples were divided into 259 coded pools (five mosquitoes in each) and preserved in 1.5 mL plastic tubes. The tubes were labeled, capped, and stored at−86°C until use. RT-PCR was used to detect DENV in the samples. All positive pools were confirmed by RT-PCR. The RT-PCR products were analyzed by gel electrophoresis (1.5% agarose in Tris-acetate EDTA buffer), stained with ethidium bromide, and visualized. DENV was isolated from six female *Ae Aegypti* collected from six pools (out of 259 pools). No other viruses were detected. Only five of the nine target localities had positive pools. Samples from the remaining four localities yielded negative results. Four DENV-positive mosquitoes were collected at the aquatic stages, and two were collected at the adult stage. These results show the circulation of DENV in adult mosquitoes and offspring, indicating vertical transmission of DENV. In conclusion, this study found that, in Makkah, DENV is circulating in dengue vectors with a high significance rate, suggesting the possibility of a dengue outbreak in the future; therefore, a sensitive surveillance system is vital to predict the outbreak and for early intervention and control.

## Introduction

Dengue virus (DENV) is a single-stranded RNA virus of the family Flaviviridae. It is the most prominent mosquito-borne arbovirus worldwide (1, 2). The global incidence of dengue has dramatically increased in recent decades, and it is estimated that about half of the worldwide population is now at risk of dengue fever (DF) and dengue hemorrhagic fever. DENV is mainly transmitted by *Aedes aegypti*, a mosquito species that breeds and flourishes in stagnant water containers maintained by rain or human activity (3).

Increasing mobility within and between cities and countries has led to the introduction of DENV into new areas (4). This is illustrated very clearly in the Kingdom of Saudi Arabia (KSA) as Muslims from all over the world visit the KSA for Hajj and Umrah (pilgrimage). As many pilgrims come from endemic regions, dengue has been introduced into the Hajj and Umrah regions, including three major cities: Makkah, Jeddah and Al-Madinah. Furthermore, internal factors, including ecological changes, urbanization, and human behaviors that increase breeding sites, facilitate the spread of *Ae aegypti* (5, 6). All three cities in the Hajj and Umrah regions have had major dengue outbreaks from time to time (7–9). In 2009, the Saudi Ministry of Health (MOH) reported 3350 cases of DF in the KSA and estimated the case fatality rate to be 4.6 per 1000 (8).

One of the problems of epidemiological surveillance in DF in the KSA is the underestimation of DF cases. Dengue has been described as an iceberg with a tip presenting only a fraction of the symptomatic dengue cases reported (10). This demonstrates the alarming magnitude of silent virus transmission and the weakness of the current surveillance systems (11), which are responsible for multiple outbreaks.

Entomological surveillance can help detect DENV much earlier in *Ae aegypti* than in humans (12). DENVs have been detected in *Ae aegypti* mosquitoes from endemic and epidemic regions using surveillance systems based on clinical and laboratory diagnoses. During outbreaks, such detection help identify areas with a higher rate of transmission of the DENV, allowing vector control authorities to prioritize their efforts and focus on regions where people are at the most risk of disease (13, 14).

Effective vector control (a key component for dengue control) relies on the knowledge of local DENV species distribution and vector and human behaviors that may allow mosquitoes to avoid contact with interventions. Furthermore, surveillance of DENV-infected mosquitoes provides an early warning sign for the risk of transmission in an area and indicates the predominant circulating serotype in the vector population (15). This may enable the immediate implementation of vector control measures to prevent outbreaks. A more effective approach is to detect DENV in mosquitoes before its introduction into a human host. This approach enables the immediate implementation of vector control measures to prevent outbreaks. Furthermore, this approach provides an early warning sign for the risk of transmission in an area and indicates the predominant circulating serotype in the vector population (15).

We previously conducted a longitudinal survey of *Aedes* mosquitoes in 42,981 potential *Aedes* larval breeding sites in Makkah, KSA. We found a sizeable immature population and adaptation of *Ae aegypti* to the arid climate of Makkah (6). The objectives of the current study were to screen a sample of *Ae aegypti* mosquitoes from Makkah, KSA for DENV to identify the magnitude of DENV circulation in the *Ae aegypti* mosquito population among both adult and offspring mosquitos. This information will help the concerned authorities to develop guidelines for dengue surveillance programs in Makkah based on sensitive indicators that can facilitate preparedness, alertness, and early detection of outbreaks.

## Materials and Methods

### Collection of *Ae aegypti* Mosquitoes

Between March 2016 and December 2016, 2343 houses from nine localities in Makkah, KSA, were inspected for adult and aquatic stages of DENV. According to the epidemiological surveillance of the vector-borne disease program in Makkah, these nine localities have high suspected and confirmed DF cases. The vector-borne disease program established a local protocol wherein using the locations of confirmed DF cases as center points (households), the area within a radius of 0.5 km was carefully inspected. Each house was inspected once during the study period. Adult mosquitoes were collected using either blacklight traps set up in the selected areas for 24 h and/or manually caught using hand nets or aspirators between 4 and 6 p.m. (before dusk).

For aquatic collection, larvae at different stages were collected from households with breeding sites. World Health Organization (WHO) standard entomological kits (mosquito inspection kit bags) were used to inspect and survey the mosquitoes in the aquatic stages. The sample containers were labeled and stored in a cool box at approximately 25°C. The samples were then transported to the Vector-Borne Disease Control Laboratory for species identification and further processing.

The collected larvae and pupae were reared in the vector control rearing insectary following the Food and Agriculture Organization of the United Nations (FAO) and the International Atomic Energy Agency (IAEA) Guidelines for routine colony maintenance of *Aedes* mosquito species (16, 17). Mosquitoes caught as adults and adults developed from larvae were used for DENV detection.

A pictorial key to the *Aedes* (Stegomyia) mosquitoes of East Africa and a pictorial key for identification of subfamilies of Culicidae and genera of Culicidae as well as resources from the WHO were used to identify the *Ae aegypti* (18–22). Following labelling and coding, female *Ae aegypti* were placed, five to a tube, in 1.5 mL microcentrifuge tubes and stored at −86°C until further processing. *Ae aegypti* was the only *Aedes* species identified during this survey.

### Preparation of Mosquitoes

In all, 1295 collected mosquitoes were identified as *Ae aegypti* at the National Center for Vector-Borne Disease (Ministry of Health, Jazan). These were divided into 259 pools (five mosquitoes per pool) (23), preserved in 1.5 mL microcentrifuge tubes, labelled, capped, and stored at −86°C until use. Only female mosquitoes are tested in routine arbovirus surveillance programs (12).

### RNA Extraction

A total RNA isolation kit (Promega, Germany) was used for RNA extraction. Following the manufacturer's instructions, the mosquito samples were homogenized using a mortar and pestle (mini borosilicate glass chamber length: 60 mm, pestle diameter: 9.0 mm, 3.0 mL, Fisherbrand) in 175 μL of RNA lysis solution buffer. Then, 350 μL of RNA dilution buffer was added to 175 μL of the lysate and mixed by inverting three to four times. The mixture was then placed in a heat block for three min at 70°C. After centrifugation at 10,000 rpm for 10 min, the clear lysate was transferred to a new 1.5 mL microcentrifuge tube containing 200 μL of 95% ethanol and mixed by pipetting. The mixture was transferred to a spin column inserted into a collection tube, and centrifuged for 1 min at 10,000 rpm. The collection tube was discarded, and the filter tube was placed in a new collection tube, 600 μL of RNA wash solution was added and then centrifuged for 1 min at 10,000 rpm. Following this, 50 μL of DNase incubation mix (40 μL yellow core buffer, 5 μL 0.09 M MnCl_2_, and 5 μL DNase I enzyme) was added into the collection tube, incubated for 15 min at 20–25°C, and then 200 μL of DNase stop solution was added and centrifuged for 1 min at 10,000 rpm. The filter tube was then washed twice by adding 450 μL of wash buffer under the same centrifugation conditions, followed by centrifugation for 15 s at 13,000 rpm to remove any residual wash buffer. The high filter tube was then inserted into a nuclease-free, sterile 1.5 mL centrifuge tube, and 100 μL of elution buffer was added to elute the viral RNA by centrifugation at 10,000 rpm for 1 min.

### Reverse Transcriptase-Polymerase Chain Reaction (RT-PCR)

The detection of DENV in collected vectors was performed by RT-PCR (24–29). A one-step RT-PCR-based detection method was performed as described by Lanciotti (26) using D1 and D2 primers synthesized by Integrated DNA Technology (Belgium) (Table 1) to amplify 511 bp of the viral genome. The one-step RT-PCR was performed according to the Access RT-PCR system protocol (Promega, USA). The RT-PCR products were analyzed by gel electrophoresis (1.5% agarose in Tris-Acetate EDTA buffer), stained with ethidium bromide, and visualized using the Gel Doc XR Imaging System (Bio-Rad).

**Table 1:** Oligonucleotide primers used in reverse transcription-polymerase chain reaction (RT-PCR).

**Primer**	**Sequence 5–3**	**Genome Position**	**Size in bp**
D1, Forward primer	TCAATATGCTGAAACGCGCGAGAAACCG	134–161	511
D2, Reverse primer	TTGCACCAACAGTCAATGTCTTCAGGTTC	616–644	511

### Institutional Review Board Statement

Ethical review and approval were granted by the Institutional Scientific Review Board of Faculty of Public Health and Health Informatics, Umm-Al-Qura University, Makkah, KSA (Centre of Medicine and Medical Science Research) (project #43409019).

## Results

**Figure 1:**
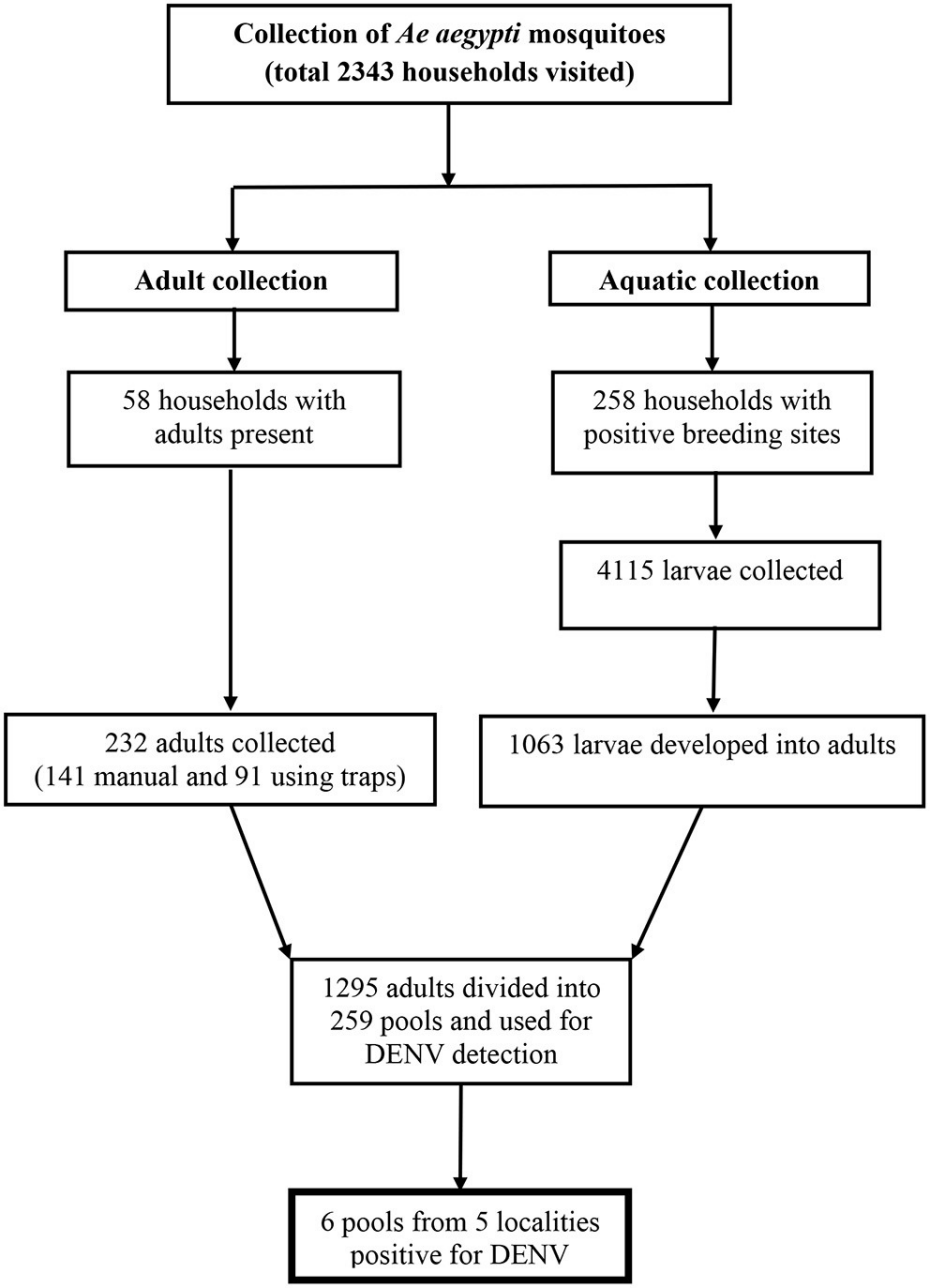
Flowchart for *Ae aegypti* collection.

The result of *Ae aegypti* mosquito collection is shown in Figure 1. In total, 1295 female adult *Ae aegypti* mosquitoes (232 caught as adults and 1063 developed from larvae into adults) were used for DENV detection.

DENV was detected in six of 259 pools (2.3%) (Table 2 and Supplementary 1).

**Table 2:** Localities with dengue cases, pools tested by RT-PCR and *Ae aegypti* larval breeding indices reported during the study period.

**Locality**	**Number of Dengue cases**	**Pools tested by RT-PCR**				**Number of inspected houses**	**House index (HI) data**		**Container index (CI) data**			**Breteau** **index** **(BI)**
		Number of Pools	Positive adult collection	Positive aquatic collection	Total positive collection		Number of infested houses	House index (HI)	Number of inspected containers	Number of infested containers	Container index (CI)	
**Al-Umarrah** ^*^	85	46	1	1	2	368	43	11.7%	509	88	17.3%	23.9%
**Al-Ghzah** ^*^	46	33	0	1	1	229	30	13.1%	345	56	16.2%	24.5%
**Al-Azizaiah** ^*^	83	38	0	1	1	353	32	9.1%	282	47	16.7%	13.3%
**Al-Maabdah** ^*^	63	30	0	1	1	272	29	10.7%	169	42	24.9%	15.4%
**Al-Sharai** ^*^	62	32	1	0	1	244	39	16.0%	237	35	14.8%	14.3%
**Al-Awali**	28	17	0	0	0	194	23	11.9%	282	31	11.0%	16.0%
**Al-Shawqiyyah**	29	26	0	0	0	243	22	9.1%	222	30	13.5%	12.3%
**An-Nawwariyyah**	32	21	0	0	0	249	20	8.0%	232	29	12.5%	11.6%
**Al-Rusayfah**	25	16	0	0	0	191	20	10.5%	229	29	12.7%	15.2%
**Total**	**453**	**259**	**2**	**4**	**6**	**2343**	**258**	**11.0%**	**2507**	**387**	**15.4%**	**16.5%**

Localities with dengue virus RT-PCR positive pool are indicated by ^*^. The house index (HI) is the percentage of houses positive for larvae; the container index (CI) is the percentage of containers positive for larvae; the Breteau index (BI) is the number of positive containers per 100 houses.

**Figure 2:**
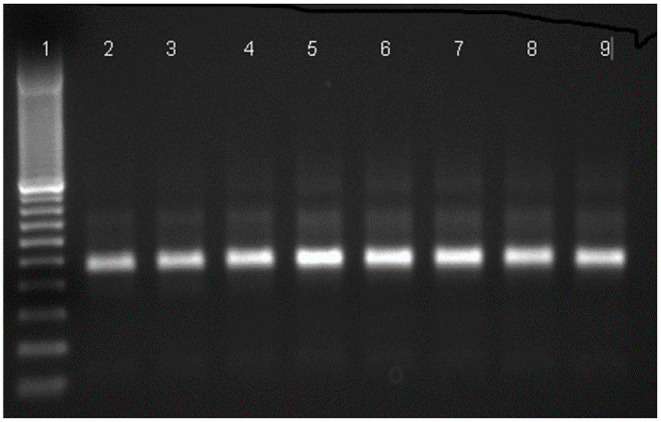
Agarose gel electrophoresis for the detection of amplified dengue virus RNA in *Ae aegypti* samples by RT-PCR using D1 and D2 primers. Lane (1) 100 bp DNA marker, lane (2-4) positive mosquito samples, lane (5-7) positive control, lane (8, 9) positive mosquito samples.

Four DENV-infected mosquitoes were collected at the aquatic stages and two were collected at the adult stage (Table 2). Results of RT-PCR for DENV are shown in Figure 2.

Five of the nine target localities (55.5%) yielded positive pools, while the other four yielded negative pools (Table 2 and Supplementary 2).

**Figure 3:**
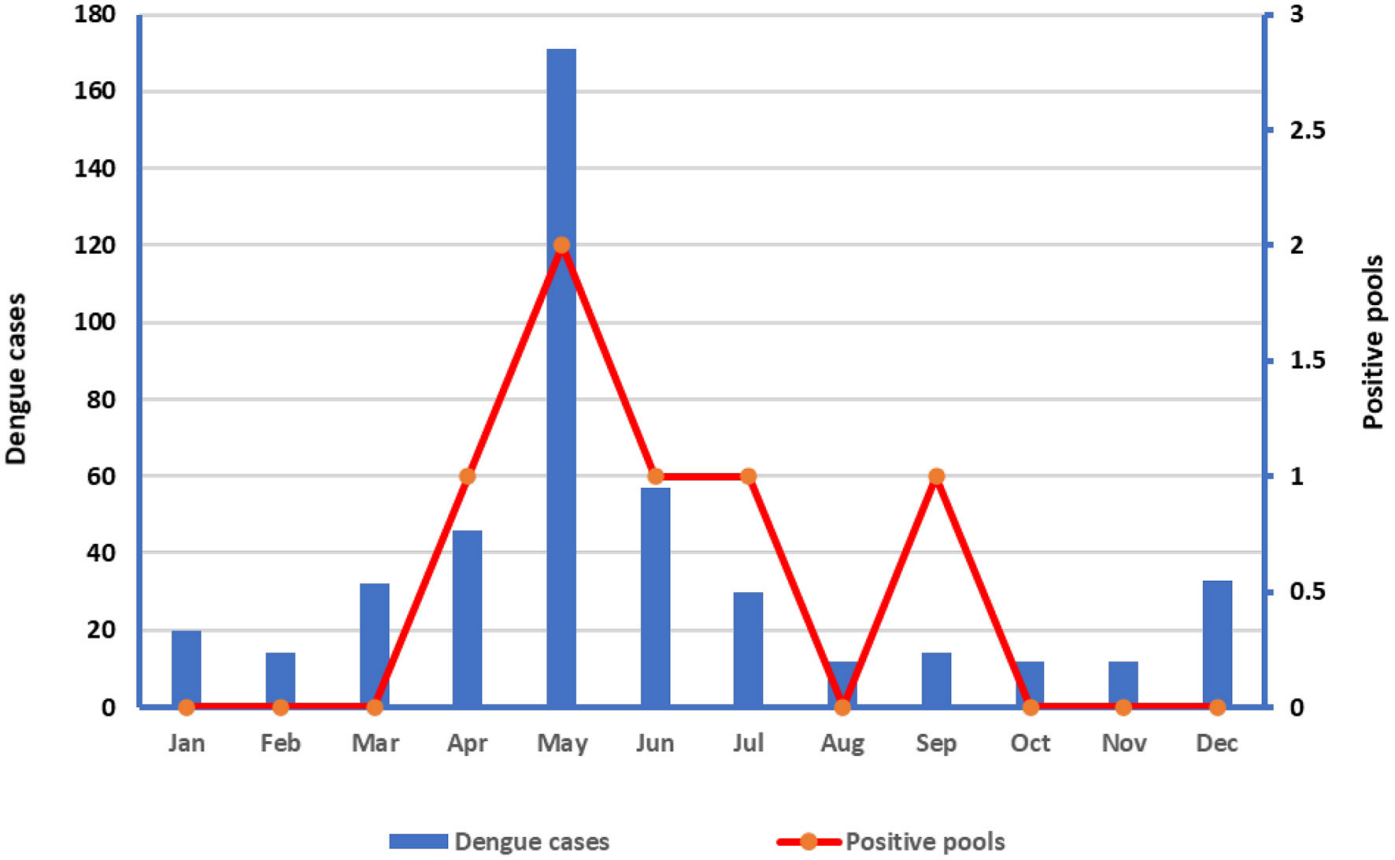
Dengue cases and dengue positive mosquito pools detected by RT-PCR.

The number of dengue cases by localities and entomological surveillance data and indices of *Ae aegypti* shown in Table 2, while the number of dengue cases in relation to positive pools is shown in Figure 3. Both the dengue cases and positive pools peaked in May (Figure 2).

## Discussion

Our results confirm that DENV is prevalent in the Makkah region, and many studies have shown that DENV-2 is the most predominant in suspected dengue cases, followed by DENV-1 and DENV-3 (7, 30–32). Furthermore, we showed the circulation of DENV in adult mosquitoes and offspring, indicating vertical transmission of DENV. Moreover, when considering the distribution of the dengue cases by month, our results showed that the cases and positive pools peaked in May (Figure 3).

Increased air travel has contributed to dengue expansion, providing the means for viremic individuals to travel from one place to another (33–37). After the infection first appeared in Jeddah, continuous surveillance revealed a rapid increase in DF cases in Makkah city and Al-Madinah, pilgrimage sites. This may be due to the availability of favorable breeding sites (e.g., water storage containers and habitats). Furthermore, our study confirmed the transovarial transmission of DENV in *Ae aegypti* vectors, supporting this well-documented finding in endemic areas worldwide (38). Moreover, this provides information on the distribution and relative abundance of potential vector populations in Makkah city. These factors are essential components in combating mosquito-borne diseases (39–41).

Detection of DENVs in mosquitoes constitutes a reliable strategy for monitoring and evaluating the strategies and interventions currently in use to comprehend the types of viruses circulating in nature and help design vector-specific strategies as indicated elsewhere (23, 41, 42). Moreover, methods for detecting DENV in infected mosquitoes are one of the essential and powerful tools for the early detection of dengue outbreaks (23, 41, 43). Herein, RT-PCR was used for DENV detection. Chow et al. (12) demonstrated that by detecting DENV in adult mosquitoes using RT-PCR, an outbreak could be predicted6 weeks in advance of the occurrence of the first human cases. Further, PCR-based screening could be used to map locations with different levels of endemicity. In combination with the Geographical Information System (GIS), PCR-based screening will provide a practical tool for vector control, enabling the identification of high-risk areas. Further, it improves the ability of epidemiological surveillance systems to anticipate outbreaks and detect silent viruses, as reported by Méndez-Galván et al. (41).

Our study is not without limitations. Sequencing and serotyping of DENV were considered, but the technology was not readily available in our setting. Moreover, due to the method of data collection, we were unable to correlate the positivity of pools in relation to dengue houses vs. neighboring houses, as shown by Urdaneta et al. (23). Nevertheless, we showed the number of dengue cases reported during the study period in relation to tested pools and *Ae aegypti* entomological indices (Table 2).

## Conclusions

In Makkah, DENV is circulating in dengue vectors with a high significance rate, indicating the possibility of a dengue outbreak in the future. This study also confirmed the transovarial transmission of DENV in the *Ae aegypti* vector. Overall, these results indicate that a sensitive surveillance system is key to predicting dengue outbreaks and facilitating early intervention and control. This information will help the concerned authorities develop guidelines for dengue surveillance programs in Makkah based on sensitive indicators that can promote preparedness, alertness, and early detection of outbreaks.

## Data Availability Statement

The raw data supporting the conclusions of this article will be made available by the authors, without undue reservation.

## Author Contributions

EA, AOB, and AOSB: conceptualization, project supervision, and funding acquisition. EA, AOB, AOSB, and Alhazmi, A.M.F: methodology. EA, SA-M, and MA-L: analysis. EA, AOSB, and SA-M: fieldwork. OD, IA-M, NM, and AA-Z: lab analysis and visualization. EA: writing—original draft preparation and project administration. AOB, AOSB, SA-M, and MA-L: writing, review and editing. All authors contributed to the article and approved the submitted version.

## Funding

Scientific Research and Revival of Islamic Heritage at Umm Al-Qura University (project # 43409019).

## Conflict of Interest

The authors declare that the research was conducted in the absence of any commercial or financial relationships that could be construed as a potential conflict of interest.

## Publisher's Note

All claims expressed in this article are solely those of the authors and do not necessarily represent those of their affiliated organizations, or those of the publisher, the editors and the reviewers. Any product that may be evaluated in this article, or claim that may be made by its manufacturer, is not guaranteed or endorsed by the publisher.
