# Supplementary material for: Detection of Dengue Virus From Aedes aegypti (Diptera, Culicidae) in Field-Caught Samples From Makkah Al-Mokarramah, Kingdom of Saudi Arabia, Using RT-PCR
Source: Front Public Health. 2022 Jun 9;10:850851. doi: 10.3389/fpubh.2022.850851 (PMC9221504; doi:10.3389/fpubh.2022.850851)
Supplement: Supplementary file 1 [file Data_Sheet_1.docx]

**Supplementary 1:** Total and positive *Ae aegypti* pools tested for dengue virus by RT-PCR.

| **Sample No.** | **No. of Pools** | **Positive Pools** |  | **Sample No.** | **No. of Pools** | **Positive Pools** |  | **Sample No.** | **No. of Pools** | **Positive Pools** |  | **Sample No.** | **No. of Pools** | **Positive Pools** |
| --- | --- | --- | --- | --- | --- | --- | --- | --- | --- | --- | --- | --- | --- | --- |
| 1 | 1 | 0 |  | 22 | 3 | 0 |  | 43 | 6 | 0 |  | 64 | 1 | 0 |
| 2 | 2 | 0 |  | 23 | 1 | 0 |  | 44 | 3 | 0 |  | 65 | 2 | 0 |
| 3 | 1 | 0 |  | 24 | 3 | 0 |  | 45 | 6 | 0 |  | 66 | 10 | 0 |
| 4 | 5 | 0 |  | 25 | 1 | 0 |  | 46 | 3 | 0 |  | 67 | 5 | 0 |
| 5 | 1 | 0 |  | 26 | 2 | 0 |  | 47 | 1 | 0 |  | 68 | 2 | 0 |
| 6 | 3 | 0 |  | 27 | 1 | 0 |  | 48 | 1 | 0 |  | **69** | **7** | **1** |
| 7 | 4 | 0 |  | 28 | 3 | 0 |  | 49 | 6 | 0 |  | 70 | 1 | 0 |
| 8 | 4 | 0 |  | 29 | 3 | 0 |  | 50 | 1 | 0 |  | 71 | 1 | 0 |
| 9 | 4 | 0 |  | 30 | 5 | 0 |  | 51 | 4 | 0 |  | 72 | 1 | 0 |
| 10 | 2 | 0 |  | 31 | 1 | 0 |  | 52 | 3 | 0 |  | 73 | 1 | 0 |
| 11 | 2 | 0 |  | 32 | 1 | 0 |  | **53** | **23** | **2** |  | **74** | **1** | **1** |
| 12 | 3 | 0 |  | 33 | 2 | 0 |  | 54 | 12 | 0 |  | 75 | 1 | 0 |
| **13** | **4** | **1** |  | 34 | 1 | 0 |  | 55 | 20 | 0 |  | 76 | 1 | 0 |
| 14 | 2 | 0 |  | 35 | 1 | 0 |  | 56 | 1 | 0 |  | 77 | 1 | 0 |
| 15 | 1 | 0 |  | 36 | 2 | 0 |  | 57 | 2 | 0 |  | 78 | 1 | 0 |
| 16 | 1 | 0 |  | 37 | 3 | 0 |  | 58 | 4 | 0 |  | 79 | 1 | 0 |
| 17 | 2 | 0 |  | 38 | 2 | 0 |  | 59 | 2 | 0 |  | 80 | 2 | 0 |
| 18 | 1 | 0 |  | 39 | 15 | 0 |  | 60 | 2 | 0 |  | 81 | 1 | 0 |
| 19 | 1 | 0 |  | 40 | 1 | 0 |  | 61 | 2 | 0 |  | **82** | **5** | **1** |
| 20 | 1 | 0 |  | 41 | 8 | 0 |  | 62 | 1 | 0 |  | **Total** | **259** | **6** |
| 21 | 2 | 0 |  | 42 | 3 | 0 |  | 63 | 2 | 0 |  |  |  |  |

**Supplementary 2:** Total number of pools in relation to the month of sampling and locality. Localities and *Ae aegypti* sample with dengue virus RT-PCR positive pool are indicated by *.

| **Months** | **Locality** | | | | | | | | | **Total** |
| --- | --- | --- | --- | --- | --- | --- | --- | --- | --- | --- |
|  | **Al-Umarrah*** | **Al-Ghzah*** | **Al-zizaiah*** | **Al-Maabdah*** | **Al-Sharai*** | **Al-Awali** | **Al-Shawqiyyah** | **An-Nawwariyyah** | **Al-Rusayfah** |  |
| **Jan** | . |  |  |  |  |  |  |  |  | **0** |
| **Feb** |  |  |  |  |  |  |  |  |  | **0** |
| **Mar** | 2 | 3 | 3 | 1 | 2 | 2 | 1 | 2 | 1 | **17** |
| **Apr** | 5 | 4 | 8* | 4 | 3 | 3 | 2 | 2 | 1 | **32** |
| **May** | 10* | 5 | 4 | 5 | 4 | 5 | 4 | 5 | 4 | **46** |
| **Jun** | 5 | 3 | 4 | 8* | 3 | 4 | 3 | 3 | 2 | **35** |
| **Jul** | 7 | 7* | 5 | 3 | 9* | 4 | 2 | 2 | 2 | **41** |
| **Aug** | 3 | 2 | 4 | 2 | 3 | 2 | 1 | 2 | 1 | **20** |
| **Sep** | 8* | 4 | 3 | 2 | 3 | 2 | 1 | 3 | 2 | **28** |
| **Oct** | 3 | 3 | 3 | 2 | 2 | 2 | 1 | 1 | 1 | **18** |
| **Nov** | 1 | 2 | 2 | 1 | 2 | 1 | 1 | 0 | 1 | **11** |
| **Dec** | 2 | 2 | 2 | 1 | 1 | 1 | 1 | 1 | 0 | **11** |
| **Total number of pools** | **46** | **35** | **38** | **29** | **32** | **26** | **17** | **21** | **15** | **259** |
| **Total positive pools** | **2** | **1** | **1** | **1** | **1** | **0** | **0** | **0** | **0** | **6** |
